# Expression of Lysophosphatidic Acid Receptor 1 and Relation with Cell Proliferation, Apoptosis, and Angiogenesis on Preneoplastic Changes Induced by Cadmium Chloride in the Rat Ventral Prostate

**DOI:** 10.1371/journal.pone.0057742

**Published:** 2013-02-22

**Authors:** Riánsares Arriazu, Esther Durán, José M. Pozuelo, Luis Santamaria

**Affiliations:** 1 Histology Laboratory, Institute of Applied Molecular Medicine, Department of Basic Medical Sciences, School of Medicine, CEU-San Pablo University, Madrid, Spain; 2 Cell Biology Laboratory, Institute of Applied Molecular Medicine, Department of Basic Medical Sciences, School of Medicine, CEU-San Pablo University, Madrid, Spain; 3 Morphology Department, School of Medicine, Autonomous University of Madrid, Madrid, Spain; Health Canada, Canada

## Abstract

**Background:**

Lysophosphatidic acid (LPA) is a phospholipid growth factor involved in cell proliferation, differentiation, migration, inflammation, angiogenesis, wound healing, cancer invasion, and survival. This study was directed to evaluate the immunoexpression of LPA-1, cell proliferation, apoptosis, and angiogenesis markers in preneoplastic lesions induced with cadmium chloride in rat prostate.

**Methods:**

The following parameters were calculated in ventral prostate of normal rats and rats that received Cd in drinking water during 24 months: percentages of cells immunoreactive to LPA-1 (LI_LPA1_), PCNA (LI_PCNA_), MCM7 (LI_MCM7_), ubiquitin (LI_UBI_), apoptotic cells (LI_APO_), and p53 (LI_p53_); volume fraction of Bcl-2 (V_F_Bcl-2); and length of microvessels per unit of volume (L_V_MV/mm3). Data were analyzed using Student's *t*-test and Pearson correlation test.

**Results:**

The LI_LPA1_ in dysplastic lesions and normal epithelium of Cd-treated rats was significantly higher than those in the control group. Markers of proliferation were significantly increased in dysplastic lesions, whereas some apoptotic markers were significantly decreased. No significant differences between groups were found in V_F_Bcl-2. Dysplastic lesions showed a significant increase of LI_p53_. The length of microvessels per unit of volume was elevated in dysplastic acini. Statistically significant correlations were found only between LI_LPA1_ and LI_UBI_.

**Conclusions:**

Our results suggest that LPA-1 might be implicated in dysplastic lesions induced by cadmium chloride development. More studies are needed to confirm its potential contribution to the disease.

## Introduction

Prostate cancer is one of the most frequently occurring cancers in male patients. The etiology of the disease is complex, and it has a multitude of potential contributing factors [1], [2]. It is thought that prostatic intraepithelial neoplasia (PIN) is the histologic abnormality most commonly associated with prostate cancer and is proposed as a precursor of invasive prostate cancer. A number of studies concluded that high-grade PIN represents the most likely precursor to invasive adenocarcinoma [3]–[8].

Lysophosphatidic acid (LPA) is a phospholipid growth factor involved in a variety of physiological and pathological process such as cellular proliferation, differentiation, migration, inflammation, angiogenesis, wound healing, cancer invasion, and survival [9]–[11]. Initially, LPA was thought to be secreted primarily by platelets during wound healing [11]; however, recently, it was believed, that LPA may be produced by many cell types such as fibroblasts and adipocytes, and it can be expressed in different tissues, including the brain, ovary, and kidney [9], [11]. Also, it is known that LPA-1 is detected in many tumors, such as those in the lung, breast, stomach, kidney, and prostate [12]. It is well established that LPA signals various events through its G protein-coupled receptors (GPCRs), namely, LPA-1 to LPA-6 [9]–[14].

LPA-1, LPA-2, and LPA-3 share about 50–57% amino acid sequence identities and form the Edg (Endothelial differentiation gene) family together with the GPCRs for sphingosine 1-phosphate. The switching expression of LPA-1 receptors is found to be associated with prostate cancer development [1], [13], [15]. LPA-4, LPA-5, and LPA-6 were classified as ‘non-Edg family’ LPA receptors and provide a new framework for understanding different LPA functions [13]. In normal tissues, LPA-1 is broadly expressed, whereas expression of LPA-2 and LPA-3 is more restricted [16]. The physiological role of LPA-4 has not been investigated [17] and LPA-5 was found to be highly expressed on cells associated with the immune system [18], and LPA-6 is a novel receptor implicated in human hair growth [13], [14].

In the case of prostate cancer, LPA is reported to induce proliferation and survival of androgen-independent prostate cancer cells [9]. Prostate cancer is the second leading cause of male deaths in the majority of Western countries [11], [19]. Information regarding the expression profile of LPA-1 in human biopsies is limited [11].

The rates of proliferation and apoptosis should be determined to have a better knowledge of the dynamism of the cell population in normal and pathological conditions and to establish the relationship with LPA-1 expression.

On the other hand, angiogenesis is a critical feature of many diseases, including cancers and their precursors [20], [21]. Angiogenesis is defined as the process leading to the formation of new blood vessels and is essential for normal growth and development [22]. Recently, LPA was demonstrated to promote ovarian cancer growth by inducing angiogenic factors [10], but this relation in prostate dysplastic lesions needs further investigation.

A useful experimental model for human prostate cancer is the rat [23]–[25], and morphological similarities between human PIN and dysplastic changes experimentally promoted in rodent prostate have been reported [26]. Our group designed an experimental model based on the administration of low doses of cadmium chloride [6]–[8], [27]. This model induces higher incidence of prostate carcinogenesis in Sprague-Dawley rats in a manner similar to those in humans.

In the present study, we estimated immunoexpression and quantification of LPA-1 in epithelial cells. Cell proliferation was determined by the quantification of proliferative cell nuclear antigen (PCNA) and the miniature chromosome maintenance (MCM7). The apoptosis was quantified using Bcl-2, ubiquitin, and p53 and by measuring the ratio of apoptotic nuclei to the total nuclei of epithelial cells using the TUNEL assay. The angiogenesis was observed by quantification of Von Willebrand factor (Factor VIII).

The aims of this study are as follows: a) to determine the LPA-1 immunoexpression in preneoplastic lesions induced with cadmium chloride; b) the evaluation of cell proliferation, apoptosis, and angiogenesis markers in these lesions; and c) to determine the correlation between LPA-1 immunoexpression and cell proliferation, apoptosis, and angiogenesis markers studied in b).

## Materials and Methods

### Ethics statement

This experiment and animal care were conducted in compliance with the guidelines established by the ‘Guide for the Care and Use of Laboratory Animals’ CEU–San Pablo University, Madrid, Spain. All rats were housed, five per cage, under controlled temperatures in a 12-h light/dark cycle with easy access to food Panlab Lab Chow (Panlab, Barcelona, Spain) and water. All the animals were euthanized using CO_2_ narcosis 24 months after the beginning of the experiment, and all efforts were made to minimize suffering. All in vivo experiments were previously published in Prostate 63: 347–357 [6], J Histochem Cytochem 54: 981–90 [8], and Hormonal Carcinogenesis IV, Springer pp. 522–528 [7].

### Care and handling of the animals and the storage of the samples

All animals used had an attending veterinarian available, and health status was monitored at least once daily. Clinical specific signs or objective measurements of organ dysfunction were monitored.

All animal rooms were checked daily, including room conditions, animals with health problems, food and water levels, and proper cage conditions. Animal rooms were kept clean, quiet, and uncluttered. Lighting conditions were adequate for the animals to behave normally and for the animal caregivers to perform their duties. The light cycle was 12-h light/12-h dark, as we previously mentioned. Ventilation was adequate to provide oxygen and remove chemical, biological, and heat waste. Room temperatures should be maintained in a range suitable for the animal species, and the animals should be protected from abrupt changes. Noise in animal rooms was minimized whenever possible. Room surfaces were constructed of material that is easily sanitized; floors, counters, and sinks were cleaned daily, and other room surfaces, including cage racks, were sanitized monthly.

Checking was done every day, including weekends and holidays, and ventilation ducts and filters were cleaned at least monthly.

### Treatment groups and experimental design

Ten adult male Sprague-Dawley rats, 30 days old at the beginning of the study, were used. Sprague-Dawley rats were randomly assigned into two groups, according to treatment (5 rats per group). Cadmium chloride (Panreac, Madrid, Spain) was added to the drinking water of the first group at a concentration of 60 ppm, during the time course of the experiment (24 months). The second group was used as control and received drinking water that was shown to be free of this metal.

After sacrifice, the organic remains from the animals and the residua of drinking water were adequately processed according to the guidelines in relation to the safety in the use of heavy metals established by the communitarian normative of the European Union.

### Tissue preparation

The prostate complex was dissected from the abdominal cavity of each animal. Dorsolateral prostate lobes were routinely examined in all groups being studied, and no dysplastic changes were detected. Then, only the ventral lobe was used in the study.

Afterward, the ventral prostate was cut exhaustively into 2-mm-width slices. The section plane was perpendicular to the sagittal axis of the gland. All specimens were fixed by immersion in 4% paraformaldehyde in phosphate-buffered saline (PBS) pH 7.4, for 24 h. Thereafter, the slices were embedded in paraffin, and the paraffin blocks were then serially sectioned at 5 µm-thickness and stained with hematoxylin-eosin or used for immunohistochemical techniques.

### Immunohistochemical methods

Deparaffinized and rehydrated tissue sections were treated for 30 min with hydrogen peroxide 0.3% in phosphate-buffered saline (PBS), pH 7.4, to block endogenous peroxidase. Antigen unmasking was performed with pepsin (15 min, Sigma, St Louis, USA). To minimize nonspecific binding, sections were incubated with serum blocking solution (Histostain® Bulk Kit, Invitrogen Corporation, Carlsbad, California) and subsequently incubated overnight with primary antibodies in a moist chamber at 4°C. Information on primary antibodies is provided in Table 1.

**Table 1:** Primary antibodies used for immunohistochemistry.

Antigen	Concentration	Supplier	Source
LPA1(EDG2)	1∶100	Novus Biologicals, Litttleton. USA	Polyclonal
UBIQUITIN	1∶200	DAKO Carpinteria, California. USA	
Von Willebrand Factor (Factor VIII)	Ready to use	DAKO Carpinteria, California. USA	
PCNA	1∶100	Biomeda, Foster City, CA	Monoclonal
MCM7	1∶50	Oncogene. Cambridge. MA. USA	
Bcl2	1∶100	DAKO Carpinteria, California. USA	
p53	1∶50	Cell Signaling, Beverly, MA	

On the second day, immunohistochemistry was performed with standard procedures using Histostain® Bulk Kit (Invitrogen Corporation, Carlsbad, California). The immunostaining reaction product was developed using 0.1 g diaminobenzidine (DAB) (3,3′,4,4′–Tetraminobiphenyl, Sigma, St Louis, USA) in 200 ml of PBS, plus 40 µl hydrogen peroxide. After immunoreactions, sections were counterstained with acetic carmine, Harris hematoxylin, or methyl green, dehydrated in ethanol and mounted in a synthetic resin (Depex, Serva, Heidelberg, Germany). The specificity of the immunohistochemical procedures was checked by incubation of sections with nonimmune serum instead of the primary antibody.

### DNA fragmentation detection

To detect the apoptotic fragmentation of DNA, a TUNEL (TdT-mediated dUTP-biotin nick end labeling) technique (Boehringer Mannheim) was used [6], [28]. This method involves the insertion of labeled nucleotides into broken ends of DNA strands. A brief description of the method follows: sections were deparaffinized and rehydrated and then incubated with proteinase K (10 µl/ml in TRIS/EDTA pH 8) for 30 min at 37°C for digestion of nuclear proteins. Endogenous peroxidase was inactivated with 0.3% hydrogen peroxide in distillate water during 30 min. The sections were incubated for 1 hr at 37°C in the TdT (terminal deoxynucleotide transferase) mixture with addition of labeled nucleotides (2∶1), and after that, they were incubated in converter POD during 30 min at 37°C. The reaction was detected using 0.1 g DAB in PBS (200 ml), plus 40 µl hydrogen peroxide. After the TUNEL technique, sections were counterstained with acetic carmine. All slides were dehydrated in ethanol and mounted in Depex (Serva, Heidelberg, Germany).

### Quantitative evaluation of LPA-1, cell proliferation, apoptosis, ubiquitin, and p53

The percentages of LPA-1–immunostained nuclei (LPA-1 labeling index, LI_LPA1_), PCNA-immunopositive nuclei (PCNA labeling index, LI_PCNA_), labeled apoptotic nuclei (apoptotic labeling index, LI_APO_), ubiquitin-immunostained nuclei (UBI labeling index, LI_UBI_), and p53-immunostained nuclei (p53 labeling index, LI_p53_) were calculated in each selected section for control rats, nondysplastic acini of cadmium-treated (Cd-treated) rats, and dysplastic acini of Cd-treated animals, using the following formula: number of labeled nuclei×100/total number (labeled + unlabeled) of nuclei [6], [8], [27], [29].

Measurements were performed using a NIKON Eclipse E400 microscope, with a NIKON Digital Camera DXM1200; ten digital images were acquired for each slide. For counting the number of cells, freeware ImageJ v1.45 was used. Software was downloaded from NIH web site (http://rsb.info.nih.gov/ij).

In all cases (LPA-1, PCNA, MCM7, ubiquitin, and p53), nuclei were considered positive regardless of staining intensity. Apoptotic nuclei were considered positive when the stain was uniform and intense.

### Quantitative evaluation of Bcl-2 immunostaining

To quantify the immunoreactivity of Bcl-2 protein, its volume fraction (V_F_) was measured and expressed as percentage of immunostained epithelium. Estimation of the V_F_ was performed using the ImageJ v1.45 program. The selected fields were photographed, thresholded, and binarized, and the percentage of epithelial area was automatically measured by the program to obtain the V_F_ [7]. The V_F_ of Bcl-2 immunoreactivity was estimated in ventral prostates from the control and experimental animals to ascertain if treatment changes the total amount of Bcl-2 immunostaining in the epithelium. This estimation was measured in each selected section from control rats, nondysplastic acini of Cd-treated rats, and dysplastic acini of Cd-treated rats.

### Quantitative evaluation of length of microvessels (L_V_MV)

The length of microvessels per unit of volume (L_V_MV/mm^3^) of prostate tissue was evaluated in normal acini of control rats, nondysplastic acini of Cd-treated rats, and dysplastic acini of Cd-treated rats. The stromal compartment was considered as the reference space. The Factor VIII vascular profiles immunostained to be eligible for counting were those sampled by the dissector frame and fulfilling the Sterio rule [30]. The L_V_MV was a calculated by the formula: L_V_ = (2×_Q-)/_A, where Q- = number of immunopositive vascular profiles and _A = total area sampled, that is, area of dissector frame (1482 µm2) multiplied by the number of selected frames [31]. These measurements were performed using the CAST-GRID program (Stereology Software Package, Silkeborg, Denmark).

### Statistical analysis

Statistical evaluation was performed using GraphPad Prism5 (La Jolla, USA). Data were presented as means ± standard deviations (SDs). The differences between groups were evaluated using Student's *t* test for parametric data and Mann-Whitney U test for nonparametric data. The correlation study was performed using the Pearson correlation test.

## Results

Dysplastic changes in the acinar epithelium of the ventral prostate were detected to the end of the experiment in all rats treated by cadmium (5 rats).

### Qualitative results

**Figure 1:**
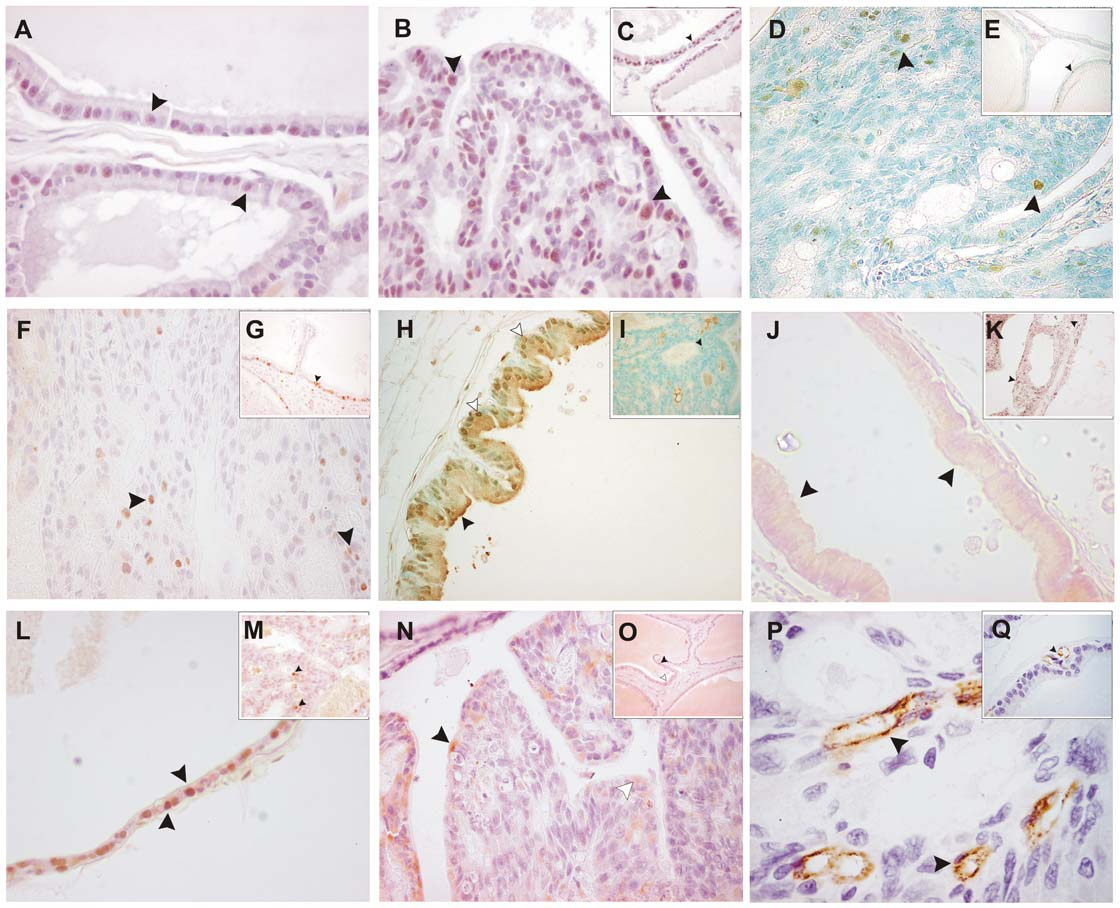
Immunoexpression detection (X400). **A**: Immunoreactive nuclei for LPA-1 are observed in the epithelium (arrowheads) of control rat. **B,C**: LPA-1 immunostained dysplastic acini (B) and nondysplastic acini (C) from a cadmium-exposed rat. Many LPA-1–positive nuclei are observed (arrowheads). The number of immunoreactive nuclei observed in B and C is higher than that in A. **D,E**: PCNA immunoexpression in dysplastic (D) and nondysplastic acini of Cd-treated rats (E). Positive nuclei are detected (arrowheads); nevertheless, dysplastic acini show a huge number. **F,G**: The positive MCM7 nuclei (arrowheads) are evident in dysplastic (F) and nondysplastic acini (G). **H,I**: Ubiquitin expression, in H control acini has immunopositive expression in the cytoplasm (black arrowhead) and in the nucleus (white arrowheads). The nucleus interior is not uniform. Dysplastic lesions (I) present positive nucleus for ubiquitin (arrowheads). **J,K**: The localization of Bcl-2 immunoreactivity is similar in normal and dysplastic epithelium. Normal acini of Cd-treated rats are homogeneous in the cytoplasm (arrowheads in J). Dysplastic acinus shows mainly granular immunoreactivity in the apical border of the epithelium. **L,M**: DNA fragmentation test. Control acinus with abundant positive nuclei (arrowheads). Dysplastic acini show few apoptotic nuclei (arrowheads). **N,O**: p53 immunoreactive nuclei (black arrowheads) and cytoplasm (white arrowheads) cells are detected in the dysplastic lesions (L) and in normal epithelium (O). **P,Q**: Endothelial positive cells to FVIII are recognized in dysplastic stromal tissue (P) and normal acini (Q). Sections counter stained: Harris hematoxylin (A,B,C,P,Q), methyl green (D,E,H,I), and acetic carmine (F,G,J,K,L,M,N,O).

The expression of LPA-1 was detectable in the cytoplasm and nucleus (Figs. 1A, 1B, and 1C). Some interstitial cells also stained slightly, but the intensity was qualitatively weaker than that in epithelial cells.

Nuclear immunoreactivity to PCNA (Figs. 1D, 1E) and MCM7 (Figs. 1F, 1G) was detected in epithelial cells from the prostate acini throughout all the groups studied. PCNA immunostaining was also remarkable in the acini with dysplastic changes (Fig. 1D), as well as MCM7 (Fig. 1F).

Ubiquitin expression was observable in the cytoplasm and in the nucleus of the epithelial cells, mainly in the peripheral acini (Figs. 1H, 1I). Dysplastic acini showed nucleus immunostaining for ubiquitin, but no cytoplasm staining was detected (Fig. 1I).

Immunoreactivity to Bcl-2 protein was observed in control and Cd-treated rats. It was granular and expressed in the apical border of the epithelium in the control group and nondysplastic acini. In dysplastic glands, Bcl-2 immunostaining was more widely extended throughout all the cytoplasm, although it was more prominent near the lumen of the cribriform structures (Fig. 1J, 1K).

Apoptotic nuclei were observed in epithelial cells from the control group and treated animals (Figs. 1L). Scarce apoptotic nuclei were also visualized in dysplastic lesions (Fig. 1M).

Immunoreactivity to p53 was detected in cytoplasm and nuclear epithelial cells from the prostate acini throughout all the groups studied. Nuclear p53 immunostaining was also remarkable in the acini with dysplastic changes (Fig. 1N, 1O).

Microvessels immunostained to Factor VIII were observed in both control and Cd-treated rat. More capillaries were apparently seen in both dysplastic acini (Fig. 1P, 1Q).

### Quantitative results

**Figure 2:**
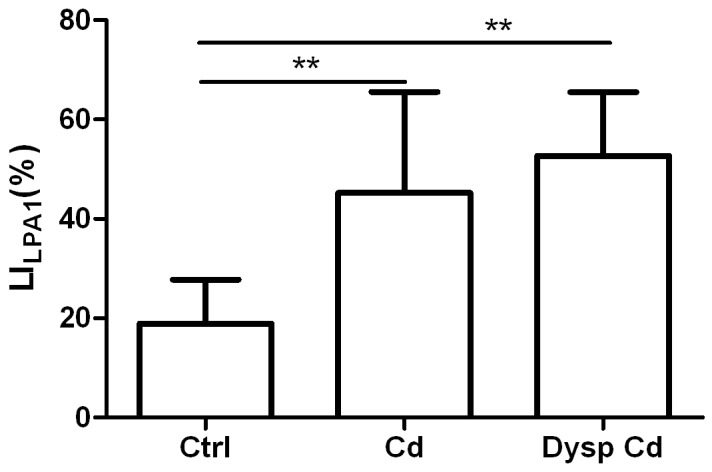
Bar diagram indicating the mean ± SD of the LI_LPA1_ in normal acini of control rats (Ctrl), normal acini of Cd-treated rats (Cd) and dysplastic acini of Cd-treated rats (Dysp Cd). Lines and asterisks indicate statistically significant differences (^**^p<0.01).

**Figure 3:**
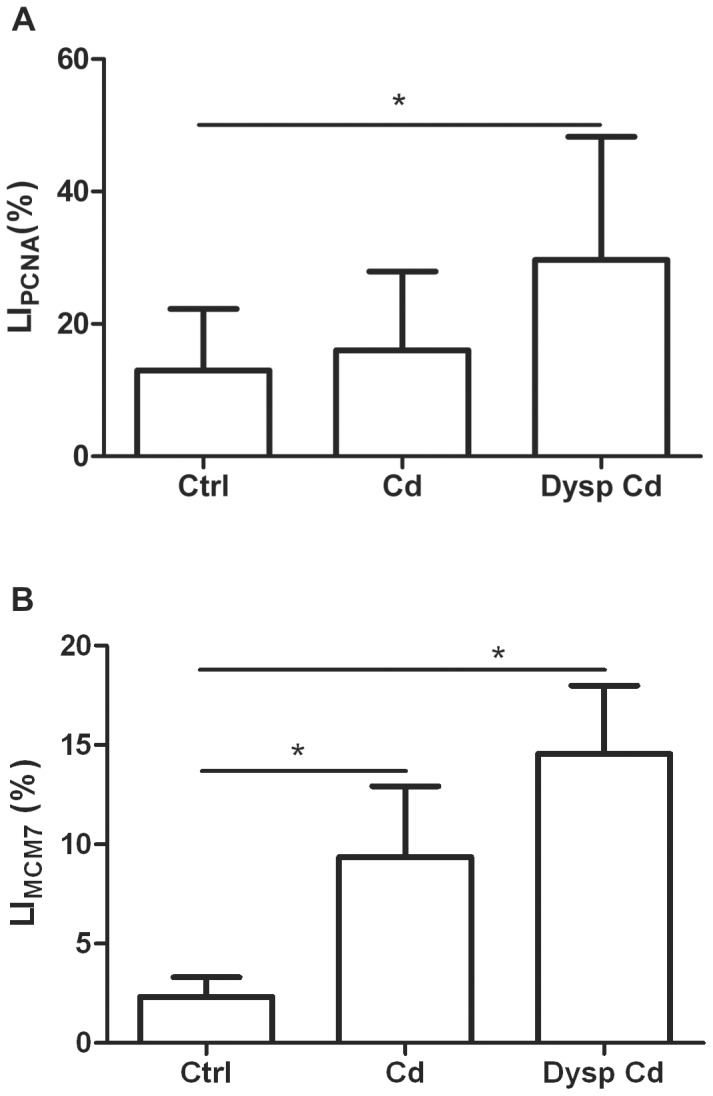
Bar graphs of the values of LI_PCNA_ (A) and LI_MCM7_. Results are expressed as mean ± SD. Lines and asterisks indicate statistically significant differences (^*^p<0.05).

A significant increase for LI_LPA1_ (p<0.01) in the normal acini and dysplastic acini of Cd-treated rats was detected in comparison to the control acini (Fig. 2). The LI_PCNA_ was significantly increased in dysplastic lesions (p<0.05), whereas the control acini and cadmium nondysplastic acini did not show significant variations (Fig. 3A). LI_MCM7_ was significantly increased in the animals exposed to cadmium (normal acini and dysplastic acini) in comparison to control animals (Fig. 3B).

**Figure 4:**
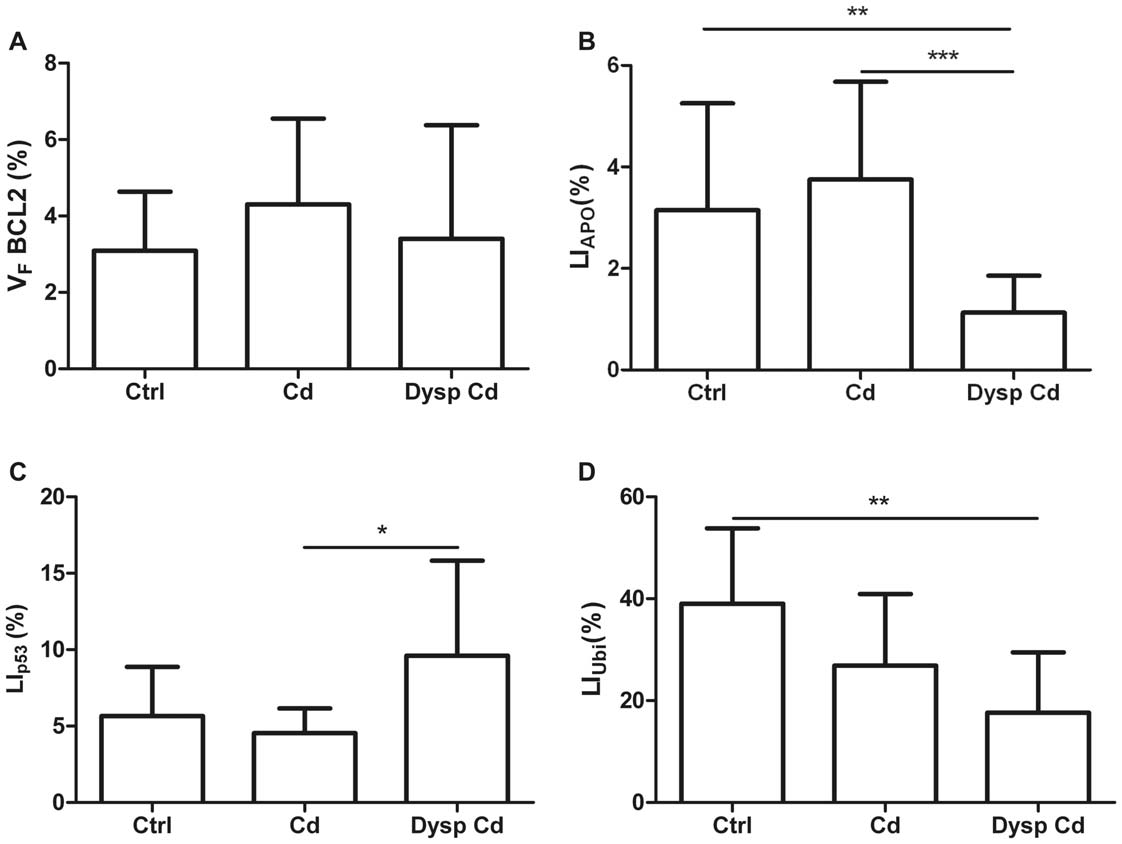
Mean ± SD of VFBcl-2 (A), LI_APO_ (B), LI_p53_ (C), and LI_UBI_ (D). Bars showing lines and asterisks on top of the error bars differ significantly (^*^p<0.05, ^**^p<0.01, ^***^p<0.001).

There was no significant difference in the V_F_ Bcl-2 between the control and cadmium-treated animals (Fig. 4A). A significant decrease of LI_APO_ was observed in dysplastic acini of rats treated with cadmium compared with the control (p<0.01) or Cd-acini (p<0.001), but any difference between the control and normal-acini of Cd-treated group was not detected (Fig. 4B). For LI_p53_, no significant differences between the normal epithelium of the control and Cd-treated rats were observed; however LI_p53_ of dysplastic acini of rats treated with cadmium was significantly increased (p<0.05) compared with cadmium normal acini (Fig. 4C). For LI_UBI_, a significant decrease (p<0.01) was observed in dysplastic acini compared with the control and normal cadmium acini (Fig. 4D).

**Figure 5:**
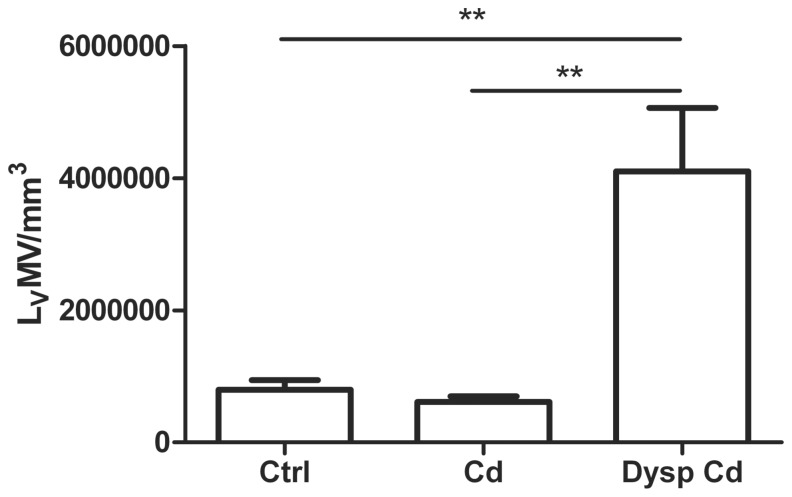
Bar diagram showing the mean ± SD of the length of microvessels per unit of volume (L_V_MV/mm^3^). Statistical differences are indicated by lines and asterisks on top of the error bars (^**^p<0.01).

The density of microvessel length (L_V_MV) did not differ considerably among control and normal acini of Cd-treated group (Fig. 5). Nevertheless, a significant increase of L_V_MV (p<0.01) in dysplastic acini was identified (Fig. 5).

When the relationship between LI_LPA1_ and proliferative, apoptotic, or angiogenesis markers in dysplastic acini of Cd-treated rats was investigated, statistically significant correlations were found only between LI_LPA1_ and LI_Ubi_ (Table 2).

**Table 2:** Pearson correlation coefficient of LPA-1 with proliferate, apoptotic, and angiogenesis markers in dysplastic acini of Cd-treated rats.

	PCNA	MCM7	Ubi	Bcl2	Apo	p53	FVIII
r	0,813	0,681	−0,919	−0,130	−0,650	0,728	0,748
Sig.	0,095	0,206	0,027	0,836	0,235	0,163	0,146

r: Correlation, Sig.: significance

## Discussion

Animal and occupational studies have strongly suggested that cadmium is carcinogenic to the prostate [2], [32]–[35]. Waalkes et al. (1989) [35] demonstrated a dose-response relation in a rodent model when tumors were induced by injecting cadmium subcutaneously. Our experimental model based on the administration of low doses of cadmium chloride induces higher incidence of prostate carcinogenesis in Sprague-Dawley rats in a manner similar to that of humans [6]–[8], [27].

In the present study, we first demonstrate that LPA-1 is expressed in dysplastic acini of Cd-treated rats. LPA-1 regulates cell proliferation, survival, angiogenesis, or migration [9]–[11]. In our study, we found that expression of LPA-1 (LI_LPA1_) was significantly higher in dysplastic acini compared with benign tissue.

Cell proliferation was studied by the expression of PCNA and MCM7. A significant difference of LI_PCNA_ in dysplastic acini was observed, as previously described by authors [8], [27], [36]–[38]. PCNA is a nuclear protein that plays a significant role in DNA replication. PCNA is a good marker for tumor growth and prognosis [20]. The miniature chromosome maintenance (MCM) complex is a group of proteins that are essential for DNA replication licensing and control of cell cycle progression from G1 to S phase. Recent studies suggest that MCM7 is overexpressed and amplified in a variety of human malignancies. Most of these studies used MCM7 as a proliferation marker to compare with proliferating cell nuclear antigen (PCNA) or Ki-67 [39]–[41]. As PCNA, LI_MCM7_ was significantly incremented in dysplastic acini; the nondysplastic acini of Cd-treated rats showed a significant increase. MCM7 is expressed in more cells than the PCNA because it is expressed in cells licensed to proliferate in addition to those that are already proliferating [40].

Not significant variation in V_F_Bcl-2 between controls and Cd-treated rats was detected, in contrast to those observed in other studies [27]. Bcl-2 is a small intracellular nonglycosylated protein that is able to inhibit the apoptotic pathway when overexpressed in cells [42]. By the TUNEL technique, we viewed a significant decrease in dysplastic acini in comparison to control acini and a representative increase of LI_p53_. p53 is a tumor suppressor protein that regulates the cell cycle and, thus, is involved in preventing cancer [43]. p53 protein mutation is more stable and have a longer half-life than functional p53 because it is easy to detect in cell nucleus using immunohistochemistry [44], [45]. p53 mutation allows cell replication without correction of DNA mutations and can be a contributing factor for the acquisition of apoptotic resistance in cadmium prostatic carcinogenesis, as described by Aimola P et al. (2012) [2]. Ubiquitin is a protein implicated in extra-lysosomal proteolysis and importantly associated with apoptosis [46]. LI_UBI_ decreased significantly in dysplastic lesions; this reduction is implicated with an apoptosis diminution, in relation to the results that we observed.

There is a remarkable increase of the L_V_MV on dysplastic lesions induced by cadmium chloride in comparison with controls and normal epithelium of Cd-treated rats; this might be related to the increase of angiogenesis indicated in cancer and preinvasive lesions by other authors [47], [48]. Angiogenesis is an important factor in growth and progression of solid neoplasms. Tumor metastasis is also angiogenesis dependent; new capillaries supply a doorway for entry of metastases to the circulatory system [20], [21].

When correlation between LI_LPA1_ with the other markers were studied in dysplastic acini of Cd-treated rats, only a significant negative correlation were observed with LI_UBI_. Nevertheless, a positive correlation with LI_PCNA_, LI_MCM7_, LI_p53_, and L_V_MV and a negative correlation with LI_APO_ and V_F_Bcl-2 were found. It can be possible that LPA-1 regulates cell proliferation and apoptosis through ubiquitin.

In summary, our study showed an immunoexpression increase of LPA-1 receptor in normal and dysplastic acini of Cd-treated rats, an increment of cell proliferation, a decrease of apoptosis, and an increase of angiogenesis in dysplastic lesions. Finally, it is possible that LPA-1 could attenuate ubiquitin immunoexpression and modify cell proliferation and apoptosis. However, the small sample size used in our work is a limitation to draw conclusions.

Overall, our findings support other reports that emphasize that LPA-1 is associated with prostate cancer development [13], [49]. Further studies are needed to determine the role of LPA-1 in prostate cancer.
